# NTD policy priorities: Science, values, and agenda setting

**DOI:** 10.1371/journal.pntd.0005431

**Published:** 2017-05-18

**Authors:** Ana S. Iltis, Kirstin R. W. Matthews

**Affiliations:** 1 Center for Bioethics, Health and Society, Wake Forest University, Winston-Salem, North Carolina, United States of America; 2 Department of Philosophy, Wake Forest University, Winston-Salem, North Carolina, United States of America; 3 History of Medicine, History and Cultural Studies, I.M. Sechenov First Moscow State Medical University, Moscow, Russia; 4 Baker Institute for Public Policy, Rice University, Houston, Texas, United States of America; Saudi Ministry of Health, SAUDI ARABIA

## Introduction

Efficient and effective implementation of strategies to combat neglected tropical diseases (NTDs), the cycle of poverty, and the political instability they perpetuate requires cogent public policy. Developing cogent NTD policy requires a clear agenda and set of priorities. Policymakers in local, regional, national, and international settings can set agendas and priorities independently, or they can collaborate with multiple stakeholders to determine the best way to develop effective NTD policy. Lack of coordination may mean that no priorities are adequately resourced or some areas are overemphasized for non-scientifically valid political reasons. NTD scientists and physicians should acknowledge the need for priorities and participate in policy development alongside public health experts and community representatives to increase the likelihood that NTD policies will be effective, efficient, and sustainable. In this article, we highlight value judgments relevant at the agenda-setting stage of the NTD policy process and describe why NTD researchers and physicians ought to participate in this process. Table 1 summarizes the key considerations that must inform NTD agenda setting.

**Table 1:** Ethical considerations in NTD policy agenda setting.

Engage stakeholders: affected communities, scientists, healthcare professionals
Promote collaboration
Coordinate efforts
Plan for appropriate oversight
Foster sustainability through education and research
Identify specific goals:
• Where will efforts be focused?
• Which diseases will be addressed?
• In what order will different NTDs be addressed?
• Will short-term or long-term goals be prioritized?
• How much attention will be given to prevention versus treatment?

## The importance of agenda setting

Policy agendas are not isolated. NTD policy is set within a web of priorities that compete for resources. Looking at the United Nations Sustainable Development Goals (SDGs) [1] can help us appreciate the importance of agenda setting. The SDGs include 17 goals and 169 targets aimed at addressing education, climate change, economic development, and health, among other areas, by 2030 [1]. Even with significant investment and political will, it is impossible to meet or even aggressively work toward all goals at once [2].

One SDG—Goal 3—is explicitly health related: “Ensure healthy lives and promote well-being for all at all ages” [1]. This vague goal includes 13 targets impacting maternal mortality, substance abuse, traffic-related deaths, tobacco control, and others. Within specific targets, priority setting is necessary to develop cogent policy. One health-related target (3.3) refers to NTDs:

“By 2030, end the epidemics of AIDS, tuberculosis, malaria, and neglected tropical diseases and combat hepatitis, water-borne diseases, and other communicable diseases.” [1]

Achieving this goal means that policy-makers and scientists need to understand the broader landscape of policy development and appreciate the many considerations pertinent to NTD policy. Many questions need to be addressed as part of agenda setting and policy planning. At a broad level, we must ask: How does NTD policy development, as well as the overall goal of promoting well-being, fit within the broader social agenda setting including issues of democracy, human rights, security from violence, and economic progression? Which NTDs should be prioritized, and in what order? What factors, such as economic, geopolitical, and health impact, should be considered in setting priorities? How should efforts balance concern with short-term and long-term consequences? Should the focus be limited to emerging endemic or epidemic diseases? How much attention should be paid to monitoring diseases to detect mutations that might render current NTDs more serious in the future? Are there any cultural restrictions on the types of interventions that may be pursued to prevent or treat NTDs? Should efforts be focused on prevention, treatment, surveillance, or prediction of diseases? How should these questions be answered, and by whom?

The next step in NTD policy requires clear interim goals that inform policy development, implementation, and assessment [2]. All epidemics of all NTDs cannot be ended at once, a point the WHO NTD Roadmap acknowledged [3]. Agenda setting is necessary and involves assigning value and significance to different possible outcomes and comparing them to establish how much attention will be devoted to each and when. It also entails determining the values, priorities, and goals that must be honored in meeting targets. NTD scientists and physicians can provide important insight into which NTDs ought to be targeted and in what order, thereby shaping NTD policy.

## Which NTD epidemics will policy target and in what order?

Decisions about where and how to address NTDs, and in what order to combat particular diseases, are critical aspects of agenda setting. They reflect judgments about which interests, needs, and goals take precedence over others. Developing a specific agenda that can guide policy development requires stating or implying that some goals are more important than others. It can be tempting to articulate priorities vaguely to generate agreement and to avoid exposing the harsh reality that some lives and needs are treated as more important than others. Yet vagueness undermines efforts to develop clear and effective policy.

Views about why it is important to combat NTDs can influence decisions about which diseases, populations, or locations should receive attention and when. For instance, should United States efforts be more concerned about NTDs that are already present in the country or ones likely to affect US populations? Recently, US policy makers increased funding for research on Zika once it was seen as epidemic in the Americas and especially after it was detected within the US. Alternatively, should the US focus efforts to achieve important political or economic goals? This would mean focusing on places where they have strategic interests that can be advanced through health diplomacy, such as helping Gulf States create a vaccine for Middle East Respiratory Syndrome (MERS). Instead, if the primary reason for combating NTDs is concern with inequity, then priority should be given to the places and people who are the worst off, such as regions in sub-Saharan Africa. These efforts could include disseminating existing treatments for NTDs in the region. Other ways of setting priorities include focusing on places where the greatest impact can be made quickly—perhaps working with populations in the US or other high-income countries where existing infrastructure might facilitate treatment and prevention efforts. Competing views about how to choose among worthy goals lead to different policy agendas. But if choices are not made and priorities are not set, then circumstances and politics will dictate the agenda.

Related to these issues are questions about the relative significance given to developing and implementing long-term strategies to end NTD epidemics versus responding to the immediate needs of people facing NTDs. Should investments fund research to understand these diseases better and aid in predicting emerging diseases? Or should funding go towards developing vaccines and treatments—despite, in some cases, limited basic understanding of the pathogen? Alternatively, should we focus limited resources of existing preventative measure and treatments to increase access? These interventions and strategies that might benefit people suffering from NTDs in the short term might not be the most effective approach for meeting the medium- and long-term goal of eliminating NTD epidemics.

## What parameters must the NTD agenda respect?

Values that may not be easily compared and that people rank differently, such as equality, liberty, security, and prosperity, yield different priorities in health policy ([4], p.46). How will they be prioritized? In some cases, certain cultural commitments or other values may set boundaries on what may be done to combat NTDs.

If a community values equality above all else, for instance, efforts that cannot be provided to all persons are unacceptable. The NTD policy agenda must then focus on equal access. For instance, early in the cholera outbreak in Haiti, many international public health experts recommended using the limited supply of cholera vaccine to attempt to “ring fence” infections and prevent the spread of disease beyond a particular region. Government officials in Haiti rejected this plan because the small available supply of vaccine would not be nearly enough to vaccinate everyone who could benefit from vaccination. They worried unequal access might trigger social unrest [5]. Many public health experts believe that even limited vaccination could have reduced the number of cholera cases by over 10% [5]. This example highlights the way underlying values and priorities lead to different views about what ought to be done [5,6].

The spread of some NTDs might be controlled by severely restricting the freedom of movement or private property rights, but many Americans would find such violations unacceptable. For instance, the Brazilian government entered houses to spray for mosquitoes without homeowner permission to curb the Zika epidemic. In the US, privacy norms have been blamed for hindering mosquito eradication efforts because access to private homes is not widely accepted [7]. Value-laden judgments can shape the NTD agenda by establishing the parameters within which specific policies must be developed.

## Who sets the NTD agenda and how?

Determining who sets priorities is just as important as agenda setting itself. Judgments about the significance of different interests, needs, and outcomes shape NTD policy. Scientists, clinicians, and public health experts might be engaged through broad surveys or through discussions aimed at building consensus, as the National Academies of Science, Engineering, and Medicine does to develop policy recommendations. Efforts should be made also to engage affected communities through collaborative partnerships with researchers, funders, local partners, and policy makers [8]. The extent to which different stakeholders are represented reflects views about whose perspectives are valuable and can influence the agenda. If some views are “overrepresented” by advocates while others receive little attention, will the imbalance be addressed and, if so, how? Furthermore, to be effective, NTD policy requires collaboration, coordination, and oversight among local, state, and the national government. This is even more complicated for international policy consensus, as different national interests must be aligned. This can be achieved by building on existing structures, such as the WHO. These collaborations are complicated and can be rife with conflict. Nevertheless, coordinating goals and efforts is vital to adopting effective, efficient, and sustainable plans toward eliminating NTDs. Sustainability also requires educating health care professionals about NTDs and cultivating young researchers’ interest and qualifications in conducting NTD research.

## Conclusion

Eliminating NTD epidemics by 2030 requires setting an agenda to meet specific and actionable NTD targets over time. If we focus on all diseases and methods simultaneously, only marginal impact can be attained. Selecting priorities will facilitate more significant achievements. NTD policy aimed at specific targets requires decisions about the balance between funding research, development, treatments, and preventative measures; which diseases to focus on, in what order, how much attention to pay to each; what constraints the agenda must respect; and who will have a voice in agenda setting. Scientists ought to acknowledge the need to set priorities to achieve goals; the importance of collaborating with public health experts, policy makers and communities to make substantial progress toward eliminating NTDs; and the inherently value-laden nature of priority setting. Only through explicitly setting priorities will effective and sustainable policies be achieved over time.
